# Influenza circulating viruses, positivity rate and risk factors for influenza associated severe acute respiratory infection during 2018/2019 winter season, Yemen

**DOI:** 10.1186/s12879-022-07090-2

**Published:** 2022-02-01

**Authors:** Mohammed Al Amad, Khaled Almoayed

**Affiliations:** 1 Yemen Field Epidemiology Training Program, Sana’a, Yemen; 2 Ministry of Public Health and Population, Sana’a, Yemen

**Keywords:** Influenza, Positivity rate, SARI, Risk factors, Yemen

## Abstract

**Background:**

The burden of seasonal influenza in conflict counties is exacerbated due to limited resource and collapse of health system. During 2018 /2019 season, two-fold increase in the incidence of influenza was reported in Yemen with 22% case fatality of severe acute respiratory infection (SARI). The aims are to analyze the influenza circulating viruses, positivity rate and risk factors for hospitalizing influenza associated-SARI.

**Methodology:**

We used a retrospective analytical study based on surveillance data. All reported patients during 2018/2019 season, fulfilling the WHO cases definition for SARI or influenza like illness (ILI), and had laboratory result from the National Laboratory were included. Influenza positivity rate was calculated, all SARI and ILI patients with positive influenza were included for further analysis by univariate and multivariate binary logistic regression. Crude and adjusted Odds ratio (AOR), 95% confidence interval and P-value < 0.05 were used for statistically significant.

**Results:**

Out of 2186 patients enrolled, 768 patients were tested for influenza viruses,: 19% were children < 15 years, 15% were ≥ 65 years, 69% males and 18% had co-morbidity with chronic diseases. Patients with SARI were 37% and 63% were ILI patients. Influenza viruses were detected in 411 (53.5%), 68% were influenza A subtype (H1N1)pdm09, 27% influenza B and 5% was influenza A not subtyped. The influenza positivity was significantly higher in SARI compared to ILI for patients < 15 years (95% vs, 66%, p < 0.001), and patients ≥ 65 years (83% vs. 56%, p < 0.002), respectively. The highest positivity for influenza type A and B reached 44% and 33% for patients ≥ 65 years and < 15 years, respectively. The risk factors for influenza-associated SARI in multivariate analysis included age < 5 [AOR 2.8] and ≥ 65 years old [AOR 3.1] compared to age 5– < 25 years, diabetes [AOR 4.7], heart diseases [AOR 3.1] and chronic respiratory diseases [AOR 5.0].

**Conclusion:**

The influenza positivity during 2018/2019 winter season was high in Yemen and varied by age distribution. Influenza subtype A (H1N1) pdm09 was the predominant and co circulated with influenza B. An influenza vaccination program for the risk group is necessary. Strengthening lab capacity to detect respiratory pathogens and further prospective study for more comprehensive picture are recommended.

## Background

Influenza is a respiratory diseases caused by influenza viruses. Type A and B are responsible for 3 to 5 million cases of severe acute respiratory infection (SARI) and 290,000 to 650,000 deaths per year [1]. The severity of influenza viruses is mainly due to exacerbations of some underlying conditions or due to bacterial co-infection and secondary infection that synergize influenza viruses and leading to severe complication, e.g. respiratory distress syndrome (ARDS), respiratory failure and death [2–4]. The burden of SARI in conflict and crisis counties is high due to the limited access and collapse of health system [5]

Yemen has started SARI surveillance at the end of 2010 by establishing two sentinel sites; one site was in Sana’a city and one was in Aden city. During 2010 to 2016, many studies on SARI patients have shown influenza positivity rate between 5–10% and 10% case fatality rate [6–8].

The electronic disease early warning system (eDEWS), due to the war and limited access to health facilities has been strengthen to function as integrated system for all infectious disease since the end of 2016. It has covered all functional healthcare facilities and became the main source of data in the county [9–11]. Almost 7132 and 94,377 of SARI and ILI cases have been reported in 2017 [11]. Increase SARI during influenza season lead to overload hospitalization and admission to intensive care units [12]. The management of SARI patients in Yemen is challenging, only 49% of healthcare facilities are functioning and the majority of these healthcare facilities has shown to be poorly equipped to manage this type of infection [13–15]. During 2018/2019 season, two-fold increase in the incidence of influenza with 22% case fatality rate among influenza associated SARI has been reported by previous study [16].

Thus, interventional prevention against seasonal influenza such vaccination as recommended by World Health Organization (WHO) [17], could help to reduce SARI incidence, hospitalization and case fatality.

Identification the type of influenza viruses as well as risk factors for influenza hospitalization are the first steps for such intervention. The aims are to analyze the influenza circulating viruses, positivity rate and risk factors for hospitalizing influenza associated SARI.

## Methods

### Study design

A retrospective analytical study based on secondary data of influenza surveillance. All SARI and ILI patients with positive influenza result were included for further analysis for the risk factors of influenza associated-SARI.

### Study population

All patients enrolled through ILI and SARI surveillance, with data collected by surveillance staff as a part of the epidemiological surveillance during 2018/2019 season (November 2018–February 2018) and had laboratory test result for influenza viruses.

### Case definitions

The surveillance team used WHO's case definitions for SARI and ILI. A SARI case defined as any patient presenting with acute respiratory infection, had a history of fever or measured fever of ≥ 38 °C within the previous 10 days and requiring hospitalization. An ILI case is defined as an outpatient presenting with acute respiratory infection including, history of fever or measured fever of ≥ 38 °C within the previous 10 days [18, 19].

### Study sites

Public and private hospitals covered by influenza surveillance, located in cold climate governorates including; AL Amanah, Albydaa, Al Mhweet, Amran, Dhmar, Hajah, Ibb, Saada, Sana,a and Taiz governorates were included in this study.

### Data collection

An investigation form was used for collecting data from adult patients or from parents in case of children. Demographic data; date of onset, clinical symptoms at admission such as history of fever or measured fever ≥ 38 °C, cough onset within previous 10 days were collected. As well as comorbidities—chronic diseases such as diabetes, hypertension, heart disease, renal diseases, respiratory diseases; asthma, chronic pulmonary disease, TB and if the case was an ILI or SARI.

### Samples

Nasopharyngeal (N) and oropharyngeal (O) swabs were collected immediately after the clinical examination. The samples were transported at 4 °C within 24 h to National Central public Health Laboratory (NCPH) along with the investigation forms. At NCPH the primers and robes provided by CDC were used for detecting influenza A and B viruses by Reverse transcription Polymerase Chain Reaction (RT-PCR).

### Statistical analysis

The data were analyzed by Epi Info version 7.2. Positivity rate for influenza was calculated by dividing the number of patients with positive influenza by total number of patients who had laboratory result for influenza. Chi-square test was used to test the differences of influenza positivity according to severity of infection and age group.

Univariate and multivariate binary logistic regression were used to calculate the crude and adjusted odds ratio, respectively with 95% Confidence Interval (CI). p value < 0.05 was considered as the cut point for statistically significant.

## Result

### Patient's characteristics

From November 2018 to February 2019, 2186 patients from ten governorates enrolled in influenza surveillance including 652 (30%) SARI cases and 1434 (70%) ILI cases. From the total 2186 patients 768 (35%) were tested for influenza viruses accounted for 43% (248 of 652) and 32% (484 of 1534) of SARI and ILI cases, respectively. Of them 19%, were children < 15 years, 15% were ≥ 65 years, 69% were males and 18% had underlying conditions; diabetes, heart diseases and renal diseases were among 10%, 6% and 6% respectively, hypertension were among 5% and respiratory diseases were among 3% of all patients.. The median age of SARI cases was 41(30–60) years compared to 35 (19–54) years for ILI cases,. Patients with SARI compared to patients with ILI were more likely to be elderly patients aged ≥ 65 years (20% vs 12%, p < 0.001) and patients with comorbidity (36% vs 8%, p < 0.001). In contrast, patients with ILI were more likely to be from children < 15 years (22% vs 13%, p = 0.004).

Table 1 shows patient's characteristics with influenza-like illness and severe acute respiratory infection during 2018/2019 season in Yemen.

**Table 1:** Characteristics of patients with influenza-like illness and severe acute respiratory infection during 2018/2019 season in Yemen

	All case		SARI cases		ILI cases		p value
	N = 768	Percent (%)	N = 284	Percent 37 (%)	N = 484	Percent 63 (%)	
Age							
< 15	143	19	38	13	105	22	0.004
15–< 25	56	7	19	7	37	8	Ref
25–< 35	118	15	38	13	80	17	0.821
35–< 45	141	18	54	19	87	18	0.567
45–< 55	110	14	50	18	60	12	0.156
55–< 65	83	11	27	10	56	12	0.864
≥ 65	117	15	58	20	59	12	0.303
Sex							
M	531	69	195	69	336	69	0.825
F	237	31	89	31	148	31	
Co morbidity							
Yes	142	18	102	36	40	8	< 0.001
Diabetes							
Yes	80	10	61	21	19	4	< 0.001
No	688	90	223	79	465	96	
Hypertension							
Yes	38	5	32	11	6	1	< 0.001
No	730	95	252	89	478	99	
Heart disease							
Yes	44	6	36	13	8	2	< 0.001
No	724	94	248	87	476	98	
Renal diseases		0					
Yes	49	6	31	11	18	4	< 0.001
No	719	94	253	89	466	96	
Respiratory							
Yes	23	3	18	6	5	1	< 0.001
No	745	97	266	94	479	99	

SARI, severe acute respiratory infection. ILI, influenza like illness

### Influenza positivity rate

Influenza viruses were detected in 411 samples out of 768 tested samples that give an overall 53.5% influenza positivity rate, 59% (167/284) in SARI patients and 50% (244/484) in ILI patients. Out of 411 positive samples, 280 (68%) were influenza A subtype (H1N1) pdm09, 110 (27%) were influenza B and 21 (5%) were influenza A not subtyped. The overall positivity of influenza was significantly higher among SARI patients compared to ILI patients (59% vs 50%, p = 0.041). It also was significantly higher among SARI than ILI patients in children < 15 years (95% vs, 66%, p < 0.001), age group 45– < 55 years (60% vs. 37%, p = 0.014) and elderly patients aged ≥ 65 years (83% vs. 56%, p < 0.002). For other age groups, e.g. 15–< 45 years, slightly higher positivity among ILI patients than SARI, but no statistical difference between the two groups.

**Fig. 1:**
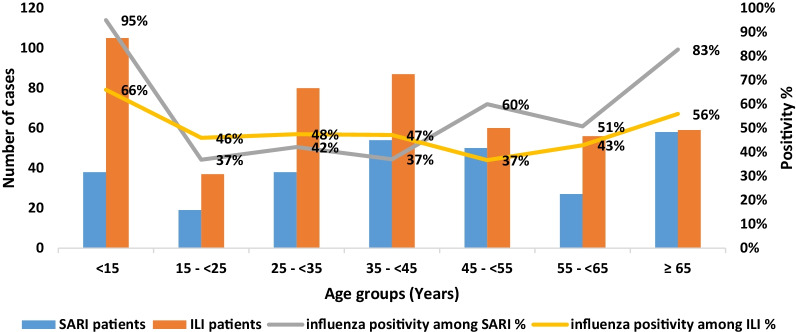
Influenza positivity among patients with influenza-like illness (ILI) and severe acute respiratory infection (SARI), by age group, 2018–2019 season, Yemen

Figure 1 shows influenza positivity rate among patients with influenza-like illness (ILI) and severe acute respiratory infection (SARI), by age group, 2018–2019 season, Yemen.

From the overall 53.5% influenza positive cases, 39% (301) were confirmed with influenza A and 14% (110) with B. The highest positivity rate of influenza A was 44% among ≥ 65 years with slight fluctuation in other age groups. The highest positivity rate for influenza B was 33% among < 15 years old.

**Fig. 2:**
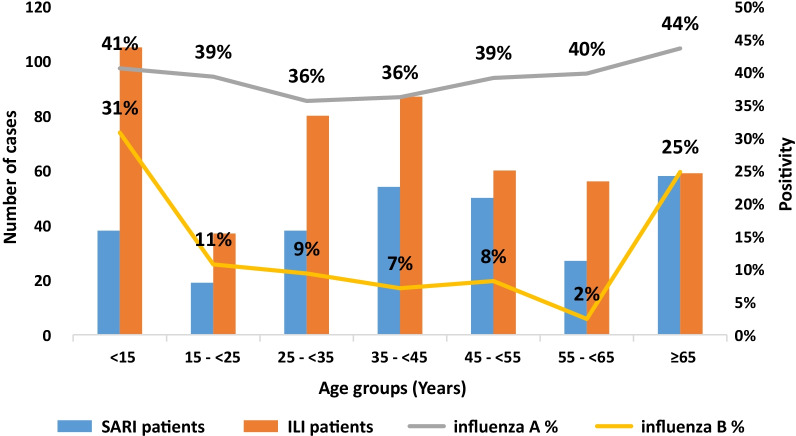
Positivity of influenza type A and B among patients with influenza-like illness (ILI) and severe acute respiratory infection (SARI), by age group, 2018–2019 season, Yemen

Figure 2 shows positivity of influenza type A and B among patients with influenza-like illness (ILI) and severe acute respiratory infection (SARI), by age group, 2018–2019 season, Yemen.

### Risk factors for hospitalizing influenza-associated SARI

From 411 patients with positive influenza result, 167 (41%) SARI patients and 244 (59%) ILI patients were included in univarate and multivariate analysis. The significant associated risk factors considered in univariate analysis were age between 45 and  < 65 (OR 2.4) and ≥ 65 years (OR 4.0) compared with 5– < 25 years, diabetes (OR = 7.0), hypertension (OR = 7.3), chronic heart disease (OR = 7.3), chronic renal diseases (OR = 2.9) and chronic respiratory diseases (OR = 6.2). The risk factors that remained independently associated with influenza-associated SARI in multivariate analysis were ages < 5 years (OR 2.8, 95% CI 1.2–6.8, p = 0.026), ≥ 65 years (OR 3.1, CI 1.2–7.8, p = 0.016) compared to 5– < 25 years old. Also diabetes (OR 4.7, 95% CL: 2.3–9.5, p < 0.001), heart diseases (OR 3.1, 95% CI 1.3–15.0, p = 0.018) and chronic respiratory disease (OR 5.0, 95% CI 1.2–19.9, p = 0.021).

Table 2 shows univariate and multivariate analysis for influenza associated SARI during 2018/2019 season, Yemen.

**Table 2:** Univariate and Multivariate Analysis for Influenza Associated Severe Acute Respiratory Illness, 2018/2019 Season, Yemen

Variables	Influenza Associated SARI	influenza Associated ILI	Univariate analysis		Multivariate analysis	
	167	244	Odds ratio (95% CI)	P value	Odds ratio (95% CI)	P value
Age						
< 5	33/167 (20)	48/244 (20)	2.2 (0.9–4.9)	0.058	2.8 (1.2–6.8)	0.026^a^
5–< 25	11/167 (7)	35/244 (14)	Ref		Ref	
25–< 45	37/167 (22)	79/244 (32)	1.5 (0.7–3.2)	0.317	1.9 (0.8–4.5)	0.124
45–< 65	41/167 (25)	46/244 (19)	2.4 (1.3–6.3)	0.010^a^	2.3 (0.9–5.7)	0.052
≥ 65	45/167 (27)	36/244 (15)	4.0 (1.8–9.0	0.001^a^	3.1 (1.2–7.8)	0.016^a^
Diabetes	50/167 (30)	14/244 (6)	7.0 (3.7–13.2)	< 0.001^a^	4.7 (2.3–9.5)	< 0.001^a^
Hypertension	26/167 (16)	6/244(2)	7.3 (2.9–18.2)	< 0.001^a^	1.2 (0.3–4.1)	0.790
Heart disease	26/167 (16)	6/244(2)	7.3 (2.9–18.2)	< 0.001^a^	3.1 (1.3–15.0)	0.018^a^
Renal diseases	24/167 (14)	13/244 (5)	2.9 (1,8–6.0)	0.001^a^	1.8 (0.7–4.1)	0.214
Respiratory	12/167 (7)	3/244 (1)	6.2 (1.7–22.7)	0.001^a^	5.0 (1.2–19.9)	0.021^a^

^a^Statistically Significant, SARI, severe acute respiratory infection. ILI, influenza like illness

## Discussion

In this study, we analyzed influenza surveillance data to provide insight into influenza positivity, type of influenza viruses and risk factors for influenza-associated hospitalization in the crisis country, Yemen.

Our finding showed high positivity rate for influenza among both SARI and ILI patients. This result might be due to the time of infection, which occurred during winter months when the transmission of human influenza usually reaches the highest peaks [20–24]. This result was within the range of reported influenza positivity during 2018/2019 season form Western Asia where our country is located [24].

Furthermore influenza type A and subtype A (H1N1) pdm09 were the predominant circulating viruses. This result was consistent with the result reported from some countries in Western Asia (Kuwait and Qatar) and other countries form Middle East and North Africa region during the previous two seasons 2017/2018 and 2018/2019 [20, 23–26]

An increase in number of SARI cases corresponded to the peaks of influenza activity during 2018/2019 season has been reported in neighboring countries: Saudi Arabia and Oman [24]. This was consistent with our result that showed higher positivity in SARI patients.

Out study showed varied positivity rate for influenza in SARI and ILI patients, higher positivity in children < 15 years and patient's ≥ 65 years compared to other age groups within both SARI and ILI patient. Higher positivity in patients with SARI compared to those with ILI except for age group ≥ 15 to < 45 years old. This variation has been demonstrated in many studies with some differences; study in Morocco showed highest positivity rates among ≥ 65 years of SARI patients compared to 5–15 years of ILI patients [25]. Study conducted in Egypt reported similar positivity among < 15 years, increase in the positivity in SARI and decrease in ILI patients ≥ 15 years old [27]. Other study in China revealed similar activity of influenza virus among patients < 5 years with ILI and SARI [28].

The difference might be due to the different in the size and age distribution of population under study. Equal proportion for age groups in our study with higher ages compared to large population and predominates of children < 15 years in those studies.

However, influenza B viruses were not predominant in our results and it was similar to the countries of Western Asia where our country is located [24]. Our study showed different positivity rates of influenza B in different age groups, the highest positivity was among children < 15 years, followed by patients ≥ 65 years old.

The higher positivity rate among ≥ 65 years was consistent with a study conducted in Egypt which revealed that influenza B was more common in patients aged ≥ 65 years [27]. The highest positivity rate in children might be due to the fact that even influenza B affects all age groups, children play as vectors for the spread of virus and schoolchildren are more infected [29, 30].

Out findings showed high detection rate among SARI patients 59% compared to (5%–10.9%) of previous studies conducted in Yemen during period 2011–2016 [6–8, 31, 32]. This might be due to that SARI patients in previous studies were from whole year, while patients of our study were from winter months. This result was consistent with previous study which demonstrated 23% detection rate for influenza from SARI patients during winter season compared to 2% during the rest 3 seasons of the year (p value < 0.001) [6].

The multivariate analysis of this study identified risk factors for hospitalization of severe influenza-associated SARI including extremes age < 5 and ≥ 65 years and three comorbidities: diabetes, heart disease and chronic respiratory disease. These risk factors have been founded in several studies conducted in developing and developed countries [25, 33–35]. As well as, they were within the risk group to whom WHO recommended influenza vaccination [17].

The risk of chronic renal disease is certainly the subject of a confounding effect due to the statistical association with the diabetes and heart disease.

In contest to result of study conducted in Morocco, males were found to be not associated with hospitalization of influenza associated-SARI. This might be due to equal proportion of patients according to sex in our study compared to the over-recruitment of women in that study.

There are some limitations in this study. It is based on secondary data collected from ten governorates patients co-morbidities included only the most common chronic disease in Yemen. The risk of other co morbidities such as HIV, malnourished people and pregnancy were not studied because either this information was not available in the data or the low prevalence such as HIV. Furthermore, the results for influenza B subtype and other respiratory viruses such as Respiratory *Syncytial Virus* were not available due to the shortage of laboratory kits. As well as bacterial etiology such as Streptococcus pneumoniae, *Haemophilus influenzae*, and *Staphylococcus aureus* that contributed in hospitalization of SARI [2] were not available.

Nevertheless, the current study provides information about influenza positivity and risk factors for hospitalization of influenza associated SARI in Yemen, particularly in cold climate governorates. It could help public health authorities to introduce influenza vaccine for people at high risk particular under 5 years and elderly people, diabetics, patients with cardiac diseases and respiratory diseases.

## Conclusion

The positivity rate for influenza during 2018/2019 season was high in cold climate governorates of Yemen. The positivity rate for influenza viruses among patients with SARI and ILI varied according to age distribution. Influenza Type A virus and subtype AH1N1 were predominant circulating viruses and co circulated with influenza type B. Influenza type A virus was higher among elderly SARI patients, while influenza type B virus was higher among < 15 years old. Extremes age < 5 and ≥ 65 years, underlying conditions including diabetes, cardiac diseases, chronic respiratory diseases were the identified risk factors for hospitalization of influenza associated SARI. Introducing influenza vaccine for these risk groups is highly recommended. A prospective study to assess co morbidities as risk factors for influenza associated SARI should be conducted. Strengthening laboratory capacity to detect other respiratory pathogens is highly recommended.

## Data Availability

All relevant data are presented in this paper; and more information can be provided upon reasonable request from the correspondence author.

## Abbreviations

AOR: Adjusted Odds Ratio
CI: Confidence Interval
eDEWS: Electronic disease early warning system
ILI: Influenza like illness
NCPH: National Central public Health Laboratory
OR: Odds Ratio
RT-PCR: Reverse transcription polymerase Chain Reaction
SARI: Severe acute respiratory infection
WHO: World health Organization
